# Dépigmentation cutanée cosmétique des femmes noires: résultats d’une enquête CAP à Abidjan (Côte d’Ivoire)

**DOI:** 10.11604/pamj.2016.24.159.8315

**Published:** 2016-06-23

**Authors:** Sarah Kourouma, Ildevert Patrice Gbery, Mamadou Kaloga, Elidjé Joseph Ecra, Abdoulaye Sangaré, Isidore Yao Kouassi, Komenan Kassi, Alexandre Kouamé Kouassi, Pauline Yao Yoboué

**Affiliations:** 1 Centre de Dermatologie du CHU de Treichville d’Abidjan, Côte d’Ivoire

**Keywords:** Dépigmentation cutanée cosmétique, motivations, femme noire, complications, Cutaneous depigmentation for cosmetic purposes, motivation, black woman, complications

## Abstract

**Introduction:**

La dépigmentation cutanée cosmétique est une pratique largement répandue chez les femmes noires en Afrique. Elle comporte de nombreuses complications bien décrites depuis des décennies. Cependant, les motivations des pratiquantes ne sont pas bien connues. Notre étude avait pour objectif d'appréhender les raisons et les motivations de ces femmes afin de pouvoir mener une action de communication en vue d'un changement de comportement.

**Méthodes:**

Nous avons mené une étude transversale qui a consisté en une enquête CAP (Connaissances/Attitudes/Pratiques) au centre de Dermatologie du CHU de Treichville d'Abidjan. Les données ont été analysées par les logiciels Epi Info 3.5.1. et 6.04.

**Résultats:**

Les pratiquantes étaient surtout des femmes urbaines jeunes (20-40 ans), célibataires, lettrées et professionnellement actives. La dépigmentation cutanée et ses conséquences étaient connues des femmes Cependant, elles désiraient être plus belles grâce à un teint plus clair et étaient influencées par les médias et leurs amies proches. Les complications les plus fréquemment observées étaient l'ochronose exogène et les vergetures. Les moyens de communication de proximité étaient les plus souhaités par les utilisatrices pour les aider à changer de comportement.

**Conclusion:**

L'élaboration de stratégies de communication de proximité visant un changement de comportement semble nécessaire pour enrayer le phénomène de dépigmentation cosmétique des femmes à Abidjan.

## Abstract (en)

**Introduction:**

Cutaneous depigmentation for cosmeticis purposes is a widespread practice among black African women. It has many complications that have been well documented for decades. However, the reasons of practitioners are not well known. The aim of our study was to understand the motivating reasons of these women in order to conduct a communication campaign for behavior change.

**Methods:**

We performed a cross-sectional KAP survey (Knowledge/Attitudes/Practices) at the Dermatology Department of the University Hospital of Treichville (Abidjan) Data were analyzed using Epi Info 3.5.1. and 6.04 software.

**Results:**

Practitioners were mostly young urban single, literate and professionally active women (20-40 years). Cutaneous depigmentation and its consequences were known to women, however, they thought that women with the lightest complexion were more attractive. They were influenced by media and friends. The most frequently observed complications were exogenous ochronosis and stretch marks. The local means of communication remained what essentially sustained the information needs of these women, because they help them to change their behavior.

**Conclusion:**

The development of local communication strategies for behavior change seems necessary to stop the phenomenon of cutaneous depigmentation for cosmetic purposes in black female population in Abidjan.

## Introduction

La dépigmentation cutanée cosmétique, artificielle ou encore volontaire, pratique bien connue en Afrique noire, observée aussi dans les populations noires et métissées d'Europe et des Etats-Unis, se définit comme l'ensemble des procédés visant à obtenir un éclaircissement de la peau dans un but cosmétique [1–3]. C'est une pratique essentiellement féminine en Afrique qui touche un quart à plus des deux tiers des femmes [1]. Elle est rapportée surtout dans les pays subsahariens notamment le Sénégal [4], le Mali [5, 6], le Togo [7], le Burkina Faso [8], le Nigéria [9–11], le Congo [12] et l'Afrique du sud [13]. En Côte d'Ivoire, la prévalence dans la population d'Abidjan, capitale économique, était estimée à 53% en 2008 [14]. L'éradication de cette pratique apparaît très complexe L'évaluation précise de ses déterminants socioculturels et enjeux économiques apparaît donc fondamentale pour élaborer des mesures de prévention efficaces [4]. Pourquoi cette pratique est elle si répandue dans la population féminine' Quel est le but recherché par ces femmes' Connaissent-elles réellement les conséquences de cette pratique sur leur santé' Quelle stratégie de prévention adopter pour enrayer ce phénomène' C'est pour tenter de répondre à ces questions que nous avons initié cette étude. L'objectif général est d'appréhender les causes ou raisons et les motivations des femmes qui se dépigmentent et le but recherché par cette pratique afin de pouvoir mener une action de communication en vue d'un changement de comportement.

## Méthodes

Nous avons mené une étude transversale qui a consisté en une enquête CAP (Connaissances/Attitudes et Pratiques). Elle a eu pour cadre le centre de Dermatologie du CHU de Treichville d'Abidjan, premier centre de référence des maladies de la peau en Côte d'Ivoire, pendant 2 semaines. Cette enquête a porté sur des femmes vues en consultation ou admises en hospitalisation. Ont été inclues dans l'étude toutes les femmes (âgées de plus de 15 ans) fréquentant ce centre à cause d'une dermatose liée à la pratique de dépigmentation volontaire et consentant à participer à l'étude. Nous n'avons pas inclus les femmes sous traitement présentant une dépigmentation comme effet indésirable (telle que la corticothérapie) et celles qui ont refusé de participer à l'étude. Le recueil des données a été fait à l'aide d'un questionnaire portant sur les données sociodémographiques; leur niveau de connaissances, leurs attitudes ainsi que les modalités de la pratique dépigmentante; les complications cutanées et des données concernant les moyens de communication et d'information sur cette pratique. Ce questionnaire a été administré aux patientes par entretien direct confidentiel avec le médecin dermatologue consultant. Les données recueillies ont été ensuite compilées et analysées à l'aide des logiciels EPI-INFO 3.5.1.et 6.04.

## Résultats

Durant la période d'étude, nous avons inclus 40 femmes sur les165 venues au centre et qui présentaient les caractéristiques souhaitées.13 femmes ont refusé de participer à l'étude.

### Données socio-démographiques

La moyenne d'âge était de 32,4ans avec des extrêmes de18 et 62 ans (écart-type=9,1). La tranche d'âge la plus concernée était celle de 20 -40 ans (80%des femmes). 42, 5% avait un niveau d'études secondaires et 27,5% supérieur. Les commerçantes étaient les plus nombreuses (30%), suivies des cadres moyens (27,5%). Les pratiquantes étaient surtout célibataires (57,5%). Les 2/3 (62,5%) avait au moins un enfant.87, 5% résidaient à Abidjan. La plupart des femmes (35%) habitaient des cités à loyer modéré (type maisons basses), 25% dans un appartement et 20% dans des «cours communes» (types de maisons basses répandus dans les villes d'Afrique subsaharienne, moins coûteux, avec vis à vis et douche partagée entre voisins).

### Connaissances sur la dépigmentation

La majorité (77,5%) des patientes savaient ce qu'était la dépigmentation cutanée et en ont donné une définition exacte. 67, 5% d'entre elles connaissaient les complications cutanées liées à cette pratique et en ont cité deux.

### Attitudes et motivations

La grande majorité des pratiquantes (80%) était convaincue que les femmes de teint clair étaient plus attirantes et plus belles que les femmes de teint noir. La moitié des femmes (50%) affirmaient que le but recherché par leur pratique était de« clarifier leur teint et faire disparaître les tâches pour être plus belle». La plupart (60%) affirmait être persuadée de l'efficacité des produits dépigmentants pour atteindre leur but avant de débuter la pratique. Près des deux tiers de ces femmes (57,5%) avait pris la décision de débuter la dépigmentation elles mêmes. 37, 5% reconnaissaient qu'une amie proche les y avait motivé. Seulement 5% des femmes ont affirmé avoir initié cette pratique à la demande de leur conjoint. Les ¾ des femmes (75%) ont avoué leur regret de s'être dépigmentée. 76,7% d'entre elles le regrettait à cause de l'apparition de maladies de la peau.

### Modalités de la pratique de dépigmentation

Toutes les femmes (100%) ont reconnu pratiquer la dépigmentation volontaire. La moitié (20 /40) a affirmé utiliser 3 produits dépigmentant simultanément en les« mélangeant». 22,5% des patientes ont affirmé utiliser effectivement ces produits depuis moins d'un an, 20% depuis dix ans, 15% depuis 5 ans, également 15% depuis 3 ans(avec des extrêmes de 6 mois et 20 ans). Près de la moitié (47,5%) n'ont jamais arrêté dont 68,4% «pour ne pas noircir». Toutes les femmes appliquaient ces produits chaque jour sur le corps et le visage. La plupart (47,5%) les achetaient au marché; 17,5% dans un magasin de cosmétiques et 12,5% à la pharmacie. 1/3 environ (30%) des patientes achetaient ces produits à 2000FCFA par mois (3,5 euros environs); 27, 5% à plus de 10 000FCFA et 22,5% à 5000FCFA. La majorité des femmes (87,5%) a affirmé financer elles mêmes leur achat. La quasi-totalité (90%) des pratiquantes a déclaré vouloir abandonner à cause de l'apparition des problèmes de peau.10% ont avoué qu'elles n'abandonneront pas malgré leur connaissance des complications car se trouvant plus belle qu'avant.

### Complications cutanées observées

**Figure 1:**
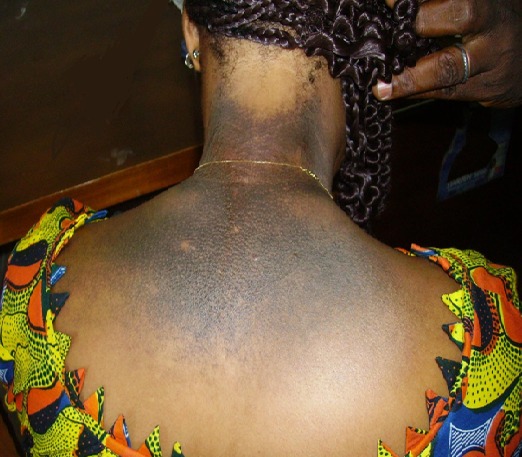
Ochronose exogène de la nuque et du dos chez une femme utilisant des produits dépigmentants

**Figure 2:**
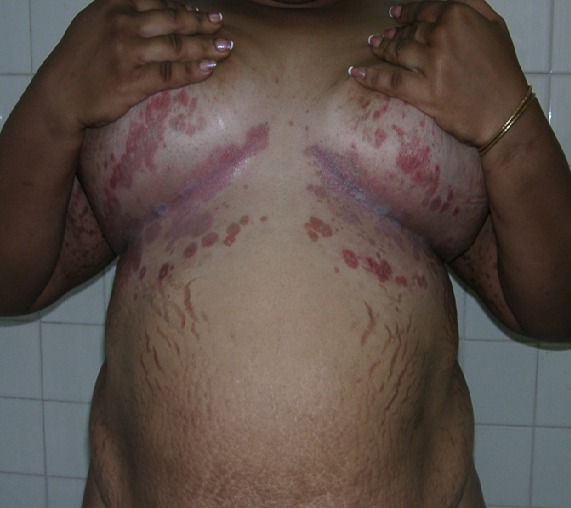
Vergetures et intertrigo sous mammaire faisant suite à la pratique de dépigmentation

Les complications cutanées constatées par les femmes elles mêmes depuis le début de la pratique étaient l'apparition de tâches noires (52,5%) et de vergetures (20%). Le diagnostic posé par le dermatologue était une ochronose exogène (Figure 1) dans 20% des cas; également 20% de vergetures (Figure 2); 17,5% de dyschromies et 10% de folliculites. Les lésions se localisaient surtout au visage (25%). Le délai d'apparition de ces lésions était ≥2 ans chez 32,5% des patientes; entre 3 et 6 mois chez 30% et de 1 an chez 15% des patientes.

### Moyens d'information et de communication

55% des patientes ont entendu parler des produits dépigmentants dans les médias audiovisuels (publicités); 15% par l'intermédiaire d'une amie proche (utilisatrice) et12, 5% au marché. La grande majorité (87,5%) a affirmé avoir déjà entendu parler des conséquences néfastes de la dépigmentation. Parmi elles 42,5% par l'intermédiaire d'une amie (ancienne pratiquante);15% dans les médias audiovisuels et 12,5% au marché. La plupart des pratiquantes (60%) pense qu'il existe d'autres moyens de sensibilisation « plus efficaces et plus accessibles »que les médias audiovisuels comme les «caravanes de sensibilisation» dans les cités, les marchés ou les salons de coiffure.

## Discussion

*La petite taille de notre échantillon (N=40) ne nous a malheureusement pas permis d'avoir une grande puissance des tests statistiques effectués. -Le biais de cette étude (dans un centre hospitalier de référence) est essentiellement un biais d'échantillonnage*.

Dans notre enquête, l'âge moyen (32,4ans) est superposable à celui de Giudice(32,6 ans) à Dakar;légèrement supérieur à celui de Pitché(28,5ans) à Lomé [15, 16] mais reste inférieur à celui de Dakar pour Morand (37,2 ans)et Raynaud (37,5 ans) [6, 17]. Cependant la tranche d'âge des 20-40 ans demeure la plus concernée dans toutes les études d'Afrique sub-saharienne [4, 8, 15–18]. La prédominance de la dépigmentation volontaire chez les 20-40 ans s'expliquerait en effet par la recherche plus active d'un partenaire pour fonder une famille, ainsi que l'ont noté les autres études [6]. La majorité des patientes résidaient à Abidjan. Ce phénomène concernerait surtout les femmes urbaines en Afrique subsaharienne [4, 16]. La plupart des femmes de notre enquête habitaient des cités à loyer modéré où vivent en grande partie la classe moyenne de la population abidjanaise. Ce qui laisserait penser à une certaine influence, un mimétisme ou effet de groupe existant dans ce type d'habitat où la vie s'organise en communautés. Les femmes lettrées sont les plus nombreuses, de même qu'à Lomé [16] ou Dakar (où le niveau n'excède cependant pas les études primaires) [4, 19] et au Burkina Faso [8]. En effet cette catégorie de femmes aurait plus accès aux médias audiovisuels et à la presse écrite donc plus influençables.

**Figure 3:**
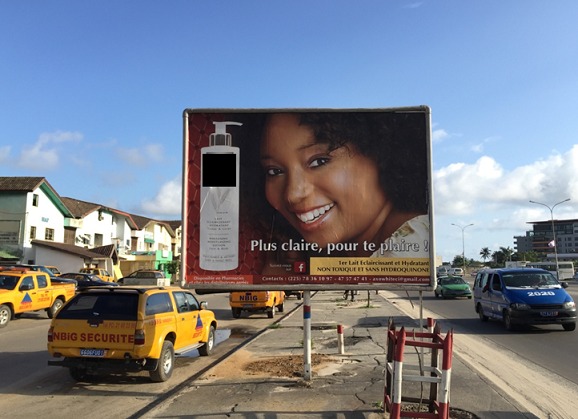
Affiche publicitaire de produit dépigmentant dans une rue d’Abidjan

La plupart des femmes dans notre étude sont commerçantes. Ce constat se retrouve dans d'autres études africaines [1, 8, 16, 18] où les utilisatrices étaient surtout des femmes exerçant une activité professionnelle comportant un contact avec une clientèle de type « commercial». Elles seraient influencées par ces contacts. La dépigmentation volontaire concerne plus les femmes célibataires dans notre enquête, comparativement à Lomé [16] et au Burkina Faso [8] mais contrairement à Dakar [4, 15] où il s'agit surtout de femmes mariées. Le désir d'être plus belle, principal but recherché par les pratiquantes, serait plus présent chez ces femmes célibataires, encore à la recherche d'un partenaire. Le phénomène contraire s'observant dans les pays où la pratique de la polygamie est officielle (Sénégal, Mali…) et où existe une rivalité entre les femmes d'un même époux. [4, 15]. Notre étude révèle que la majorité des femmes utilisatrices de produits dépigmentants ont des connaissances bien précises sur la pratique de dépigmentation et de ses conséquences néfastes sur la santé de même qu'à Dakar [4]. Il ne s'agit donc clairement pas d'une pratique par ignorance mais où la volonté, le «désir d'être plus belle» semble dominer. Les participantes de notre étude affirment que les femmes de teint clair sont plus attirantes et plus belles que les autres, convaincues qu'elles ont ainsi plus de chances de séduire les hommes. Cette observation se retrouve à Dakar [4] et Lagos [10] où les pratiquantes disent vouloir être plus belles en changeant leur teint et suivre la mode. Il faut également noter l'influence des « publicités agressives » de ces produits, omniprésentes dans les médias audiovisuels, la presse féminine africaine ainsi que dans les rues d'Abidjan (Figure 3) comme des autres métropoles africaines [1, 11, 15, 17] auquel s'ajouterait un effet de groupe ou de sous groupe chez ces femmes, résidant surtout dans des « cours communes » et cités. La plupart des femmes affirment dans notre enquête avoir pris la décision de débuter la dépigmentation elles mêmes (peut être influencées par les médias) et une grande partie reconnaît avoir été incitée par une amies proche (elle-même utilisatrice). Ce rôle de l'entourage, singulièrement des amies des utilisatrices, qui apparaissent comme des éléments incitatifs puissants, a déjà été rapporté à Dakar. Elles joueraient un rôle direct lors de l´initiation de la pratique, ainsi que pour son maintien, par l´intermédiaire de compliments fréquents [1, 4, 15]. Les ¾ des femmes ont avoué leur regret de s'être dépigmentée, la plupart à cause de l'apparition de dermatoses qui altèrent leur beauté et qui sont« visibles »par leur entourage, constituant un effet contraire au but initial recherché.

La durée de la pratique dépigmentante varie entre 6 mois et 20 ans dans notre étude, conformément aux données des études africaines [1, 7, 10, 15, 18, 19]. Le mélange de plusieurs produits dépigmentants appliqués quotidiennement sur tout le corps, pratique déjà notée [7], aurait pour but d'obtenir un «résultat net et rapide». Les utilisatrices se les procurent surtout dans les marchés selon notre enquête, de même qu'à Lomé, Dakar, Bamako, Lagos [1, 9, 16, 17, 20]. La plupart les achète à seulement 2000FCFA par mois, résultat superposable à Dakar où la moyenne est de 3000F [4, 6] et Lomé [16]. Le faible coût et l'accessibilité de ces produits pourrait aussi expliquer pourquoi cette pratique est si répandue chez les femmes, qui fréquentent beaucoup plus les marchés que les hommes, constat également fait à Lomé [16]. Ce sont les utilisatrices elles mêmes qui financent leurs achats selon notre enquête, ainsi qu'à Dakar[4] même si on note une plus grande part de financement par le conjoint à Dakar et Bamako qu'à Abidjan [1, 4]. Ceci s'expliquerait une fois de plus par la pratique de polygamie dans ces pays. Les lésions les plus fréquentes diagnostiquées par les dermatologues à Abidjan sont conformes aux données africaines avec des différences de fréquence selon les métropoles africaines [2, 5, 9, 15, 16, 21]. L'ochronose exogène, complication la plus observée dans notre étude, laisse supposer une plus grande utilisation de produits dépigmentants à base d'hydroquinone qui est la substance principalement en cause dans cette lésion [16–18]. Le délai d'apparition de ces lésions était pour la plupart ≥2 ans ainsi qu'à Lomé [16]. En effet, les effets secondaires s'observent après une période d'utilisation en fonction des concentrations des agents dépigmentants présents dans les produits [22]. On note ainsi dans notre étude également une plus grande fréquence d'apparition de ces effets entre 3 et 6 mois d'utilisation. Ceci laisse supposer une forte concentration d'actifs dépigmentants dans les produits commercialisés, surtout dans les « préparations »vendues sur les marchés, phénomène en vogue à Abidjan, sans réglementation. Ces préparations ont des compositions inconnues, véritables secrets de famille ou de commerce [22] et sont réputées auprès de la gent féminine pour leur résultat rapide. Enfin les moyens de communications de proximité dans les cités, les cours communes et les marchés sont souhaités par les pratiquantes elles mêmes car seraient plus accessibles selon elles, pour inciter les femmes à ne pas se dépigmenter et /ou à arrêter cette pratique. Ces caravanes de sensibilisation pourraient ainsi être composées de journalistes, de stars, de professionnels de santé mais aussi d'anciennes pratiquantes.

## Conclusion

Les complications cutanées de la dépigmentation volontaire semblent être bien connues des femmes utilisatrices à Abidjan; ne freinant pas pour autant cette pratique. L'intérêt d'une stratégie préventive par des moyens de communication de proximité visant un changement de comportement telle que le suggère notre étude semble nécessaire pour enrayer ce phénomène.

### Etat des connaissances sur le sujet

- La dépigmentation cosmétique est un véritable problème de santé publique en Afrique subsaharienne, concernant au moins une femme noire sur trois. Ce phénomène et ses complications graves, parfois mortelles est décrit depuis des dizaines d'années par nombre d'auteurs africains mais ne semble pas régresser.

### Contribution de notre étude à la connaissance

- Cette étude a été initiée pour tenter de trouver une solution par une méthode de prévention. Pour cela nous voulions d'abord connaître leurs attitudes et comportements sur la pratique dépigmentante, puis comprendre les motivations des femmes afin de trouver quelle méthode de prévention serait la mieux adaptée pour enrayer ce problème.
